# Successful management of proteinuria in recurrent immunoglobulin A nephropathy after deceased donor kidney transplantation: A case report

**DOI:** 10.1097/MD.0000000000036990

**Published:** 2024-01-19

**Authors:** Sehyun Jung, Seunghye Lee, Hyejin Jeon, Min Hye Kim, Jong Sil Lee, Se-Ho Chang, Hyun-Jung Kim, Hani Jang

**Affiliations:** a Division of Nephrology, Department of Internal Medicine, Gyeongsang National University College of Medicine and Gyeongsang National University Hospital, Jinju, South Korea; b Department of Pathology, Gyeongsang National University College of Medicine and Gyeongsang National University Hospital, Jinju, South Korea; c Institute of Health Sciences, Gyeongsang National University, Jinju, South Korea.

**Keywords:** case report, deceased donor kidney transplantation, immunoglobulin A nephropathy, proteinuria, renin-angiotensin system inhibitors, steroid

## Abstract

**Background:**

Immunoglobulin A nephropathy (IgAN) is the most common type of primary glomerulonephritis, and recurrent IgAN is common after kidney transplantation (KT). Owing to the differences in various biopsy protocols and follow-ups in each study, the recurrence rate varies from 9.7% to 46%. Although the relapse rates are high, there is no definitive treatment for IgAN recurrence.

**Methods:**

We present a case of successful management of proteinuria in recurrent IgAN after deceased donor KT. A 60-year-old man diagnosed with IgAN 20 years prior, who progressed to end-stage renal disease, underwent deceased donor KT 5 years prior and was admitted to our hospital with progressively increasing proteinuria.

**Results:**

The pathological examination of the kidney biopsy specimen revealed recurrent IgAN. High-dose steroid treatment was initiated, and the patient was discharged while maintaining steroid treatment. However, outpatient follow-up showed that proteinuria did not decrease while steroids were maintained. Therefore, an angiotensin receptor blocker was administered after explaining its benefits to the patient. After the addition of angiotensin receptor blocker, proteinuria continued to decrease.

**Conclusion:**

This case report highlights the importance of using renin-angiotensin system inhibitors with supportive care in cases of suspected of recurrent IgAN after KT. It also emphasizes the need to prescribe renin-angiotensin system inhibitors when steroid therapy is unsuccessful in cases of recurrent IgAN after KT.

## 1. Introduction

Immunoglobulin A nephropathy (IgAN) is the most common type of primary glomerulonephritis, and recurrent IgAN is common after kidney transplantation (KT). Recurrence rates vary between 9.7% and 46%, owing to various biopsy protocols and differences in follow-up.^[1–4]^ Despite its high recurrence rate, there is currently no well-defined treatment for IgAN. We report a case of recurrent IgAN and successful reduction of persistently elevated proteinuria in a 60-year-old man who underwent deceased donor KT (DDKT).

## 2. Case presentation

This study reports a case of IgAN recurrence in a 60-year-old male patient after KT. The patient had been diagnosed with IgAN by renal biopsy 20 years prior. Three years later, hemodialysis was initiated despite treatment with renin-angiotensin system inhibitors (RASIs). He had undergone DDKT at another nephrology center 5 years previously. Immunosuppressive therapies included prednisolone, mycophenolate mofetil, and tacrolimus. After DDKT, the patient’s blood pressure was effectively managed with a daily dose of nifedipine 40 mg. Two years prior, the patient was admitted for chest pain and was diagnosed with variant angina. The medication regimen included diltiazem 90 mg once daily, isosorbide dinitrate 20 mg twice daily, nicorandil 5 mg twice daily, and rosuvastatin 20 mg once daily. There have been no changes in the medication regimen until now. He was transferred to our hospital 3 years prior for residential reasons, and allograft function was well maintained. The tacrolimus trough levels were maintained at 5 to 10 ng/mL. Ten months prior, proteinuria was newly found, with a urine protein-to-creatinine ratio of 0.5 g/g, which gradually increased to 1.0 g/g. The patient was admitted for graft biopsy in January 2022.

At the time of admission, the patient’s vital signs were stable, and his blood pressure was 119/92 mm Hg. The patient’s body weight was 54.9 kg and height 162.1 cm. His body mass index was 20.89 kg/m^2^. Physical examination revealed normal findings, and the patient was euvolemic. Laboratory analysis revealed normal renal function, with a serum creatinine level of 1.06 mg/dL. Cytomegalovirus or BK viremia was not observed. Tacrolimus trough level was 6.7 ng/mL at the outpatient clinic before admission. The results of the other laboratory tests are presented in Table 1. No abnormal findings were observed on renal ultrasonography performed before the kidney biopsy.

**Table 1:** Serological and urinary indices.

Date	BUN (mg/dL)	Scr (mg/dL)	UPCR	TAC (ng/mL)
**February 1, 2023**	9.0	0.95	0.2	3.2
**December 21, 2022**	10.6	1.01	0.3	6.7
**September 28, 2022**	17.0	1.05	0.4	5.0
**July 20, 2022**	13.0	1.02	0.5	6.0
**June 15, 2022**	8.3	0.93	0.6	6.8
**May 11, 2022**	12.7	1.20	1.1	7.8
**April 7, 2022**	16.5	0.95	1.6	7.9
**February 9, 2022**	14.3	0.85	1.0	6.2
**January 26, 2022**	17.4	0.80	0.9	3.2
**January 20, 2022** **(on admission**)	14.7	1.06	–	–
**January 6, 2022**	8.8	0.97	1.0	6.7
**December 9, 2021**	10.9	1.00	0.4	9.4
**November 8, 2021**	11.7	0.96	0.5	3.7
**October 14, 2021**	12.0	1.01	0.2	8.5

BUN = blood urea nitrogen, Scr = serum creatinine, TAC = tacrolimus, UPCR = urine protein-to-creatinine ratio.

**Figure 1.**
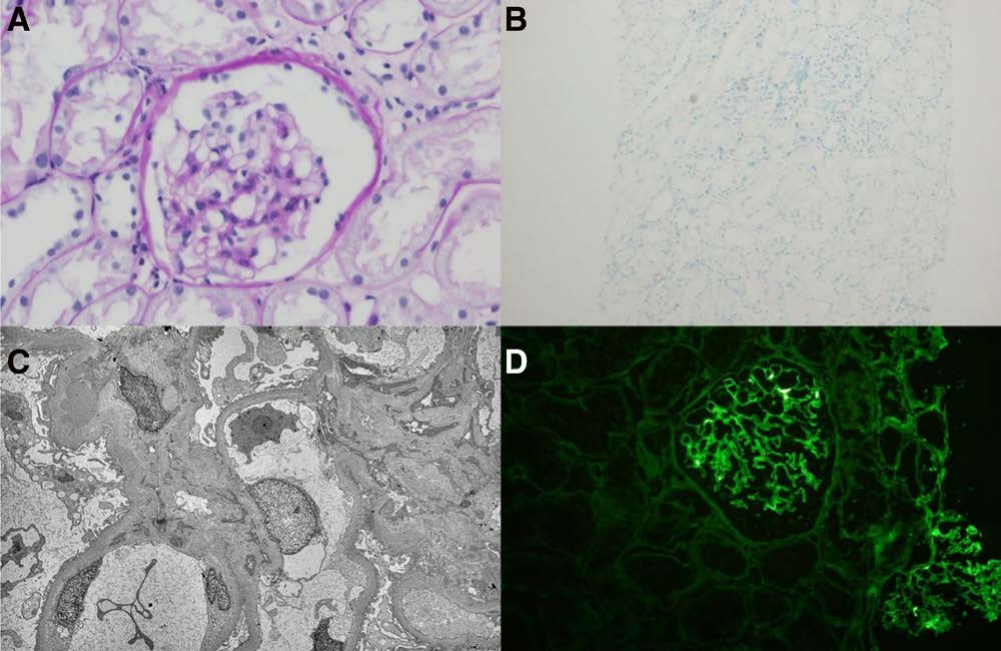
Kidney biopsy manifestations. (A) Mild increased mesangial matrix and mesangial hypercellularity are observed on light microscopy (periodic acid-Schiff-Methenamine stain, 400×); (B) C4d deposition is not observed on light microscopy (C4d stain, 100×); (C) electron microscopy shows electron-dense deposits in the mesangium; (D) deposits of IgA in the mesangium are observed on immunofluorescence (200×).

On the second day of hospitalization, a renal allograft biopsy was performed, and the renal biopsy samples contained 10 glomeruli. Four glomeruli showed global sclerosis. Light microscopy revealed a mesangial hypercellularity. C4d staining along peritubular capillaries was negative. Immunohistochemical staining revealed immunoglobulin A (IgA) deposits in the mesangium. Electron microscopy revealed small amounts of electron-dense deposits in mesangial areas. The mesangial matrix was moderately enlarged (Fig. 1). Renal biopsy results were consistent with the diagnosis of IgAN. Based on the medical history and clinical results, the patient was diagnosed with IgAN recurrence (M1, E0, S0, T0, and C0).

**Figure 2.**
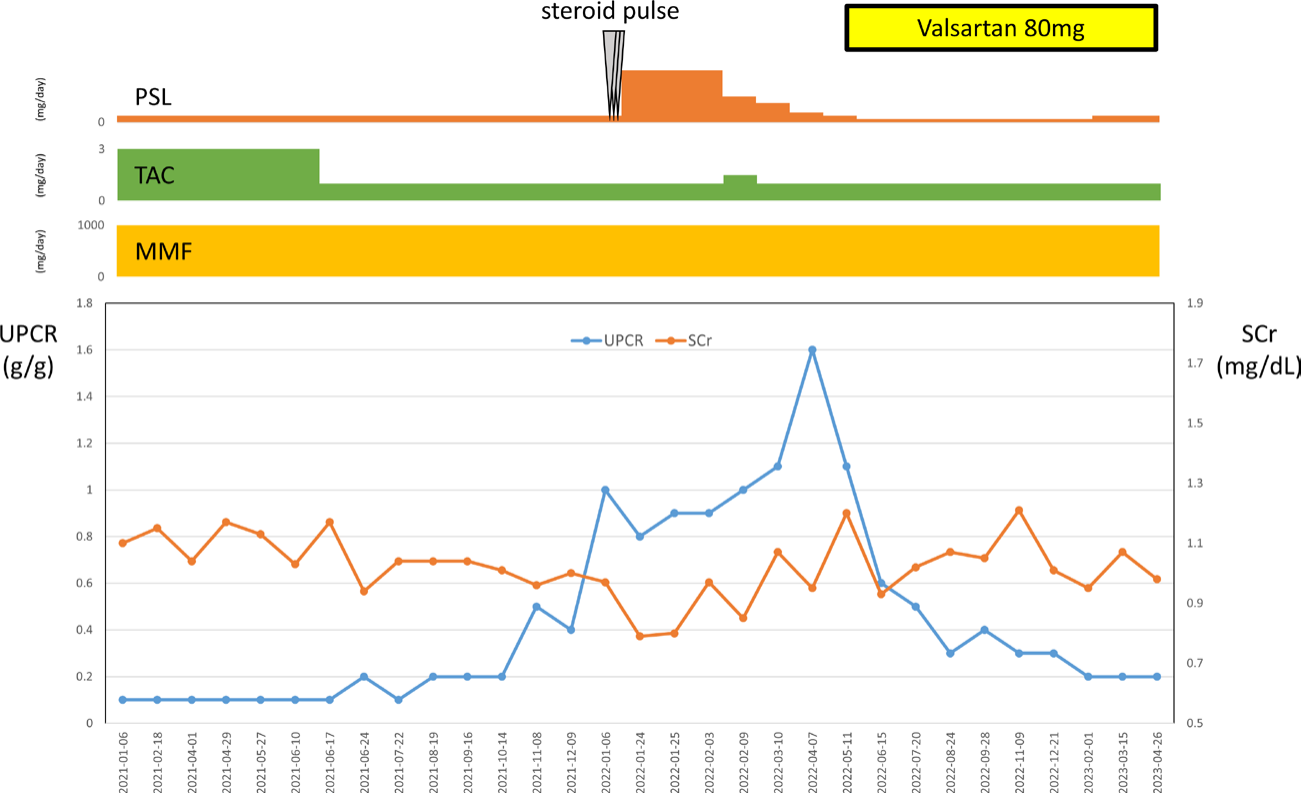
Patient’s clinical course during follow-up. MMF = mycophenolate mofetil, PSL = prednisolone, Scr = serum creatinine, TAC = tacrolimus, UPCR = urine protein-to-creatinine ratio.

Immediately after the kidney allograft biopsy, 500 mg methylprednisolone was administered intravenously for 3 days. For high-dose steroid therapy, a proton pump inhibitor was prescribed to prevent gastrointestinal bleeding, and oral trimethoprim-sulfamethoxazole was prescribed for *Pneumocystis carinii* pneumonia prophylaxis. Subsequently, 40 mg prednisolone was administered orally once daily. The patient was discharged while oral prednisolone (40 mg) was maintained. However, outpatient follow-up showed that proteinuria did not decrease while steroids were maintained. After explaining the benefits of angiotensin receptor blockers (ARBs) to the patient, 80 mg of valsartan was added on May 11, 2022. Following the addition of ARB, proteinuria continued to decrease, resulting in a 0.3 g/g urine protein-to-creatinine ratio on August 24, 2022 (Fig. 2). Outpatient follow-up showed no additional increase in proteinuria, while ARB and immunosuppressants were maintained. No prominent changes in blood pressure or increases in plasma potassium levels were observed in the patient after the addition of ARB. Three immunosuppressants (prednisolone, mycophenolate mofetil, and tacrolimus) were administered, and no dose changes were observed. Tacrolimus trough level was maintained at a therapeutic dose of 5 to 10 ng/mL.

## 3. Discussion

Recurrent glomerulonephritis is a major complication of KT. IgAN is the most common form of primary glomerulonephritis and has a high recurrence rate in transplant patients. Recurrent IgAN after KT occurs in 9.7% to 46% of patients.^[1–4]^ A recent study revealed that the cumulative incidence of recurrent IgAN was 19% at 10 years and 23% at 15 years.^[5]^ The large variance in IgAN recurrence rates in each study was due to the different racial and geographic distributions, in addition to different biopsy policies and histological evaluation techniques at different centers.^[6]^

The clinical appearance and outcomes of recurrent IgAN varies between studies. In a comparative study of recurrent IgAN using protocol biopsy, histological diagnosis was not accompanied by abnormal urine analysis, such as hematuria or proteinuria, in half of the patients.^[4]^ However, in our case, increased proteinuria was suggestive for IgAN relapse after KT.

Despite its high recurrence rate, there is currently no preventive therapy or established treatment for recurrent IgAN. Conservative treatment to prolong renal survival, such as tight blood pressure control, RASIs prescription, and avoidance of nephrotoxins, is recommended.^[7]^ Several studies have reported the positive effects of steroid treatment on recurrent IgAN.^[8–10]^

Although RASI is recommended as a conservative treatment for recurrent IgAN, doctors managing patients with kidney transplant are hesitant to prescribe angiotensin-converting enzyme (ACE) inhibitors and ARBs for the management of relapsed IgAN for several reasons. The use of RASIs causes a reduction in the estimated glomerular filtration rate, thereby making it difficult to differentiating acute rejection.^[11]^ A side effect of calcineurin inhibitors (especially tacrolimus) commonly used in patients with kidney transplant is hyperkalemia; in these patients, ACE inhibitors/ARBs can worsen the frequency and severity of hyperkalemia, which can be life-threatening.^[11,12]^ ACE inhibitors can cause or worsen anemia in transplant recipients, reducing hematocrit levels by 5% to 10%.^[13]^ Transplant renal artery stenosis usually appears between 3 months and 2 years after KT but can occur at any time,^[14]^ which is why physicians hesitate to use RASI. Transplant renal artery stenosis occurs relatively frequently after KT and is a well-known cause of posttransplant hypertension.^[15]^ Its incidence ranges from 1% to 23%.^[16]^ Based on this information, calcium channel blockers were suggested as first-line antihypertensive agents in patients with kidney transplant in a recent meta-analysis that compared the effects of different hypertension treatments on KT.^[12]^ This contributes to the reluctance of KT patients to use RASI.

Unless there are limitations to the use of RASIs in patients with KT, ACE inhibitors/ARBs can be effective in reducing proteinuria in patients with IgAN recurrence. Because of the good results reported in some studies, the use of RASIs is recommended for patients with proteinuria >0.5 g/day.^[17,18]^

While it is challenging to draw definitive conclusions based on a single case report, our study highlights the need for the use of RASIs, such as ACE inhibitors and ARB, along with supportive care when recurrent IgAN is suspected after KT and emphasizes that RASIs should be prescribed when there is no response to steroid treatment in recurrent IgAN. The limitations of this study include the possibility of delayed corticosteroid response in IgA nephropathy and the potential for spontaneous remission in IgA nephropathy.

## 4. Conclusion

In our case, we successfully managed proteinuria in recurrent IgAN after DDKT using pulse and oral steroids and valsartan. Renal biopsy should be considered if new abnormal findings are observed after KT. To date, there are no specific treatments for recurrent IgAN after KT. In this case, aggressive use of RASI was necessary for the treatment of this condition. A large randomized controlled trial is required to confirm the benefits of RASI for relapsed IgAN after KT. This case report is valuable for the treatment of recurrent IgAN after KT.

## Acknowledgments

The authors extend their sincere gratitude to the patient involved in this case and appreciate the dedication of the entire medical team contributing to this study.

## Author contributions

**Conceptualization:** Sehyun Jung, Hani Jang.

**Data curation:** Sehyun Jung, Seunghye Lee, Hyejin Jeon.

**Supervision:** Hani Jang.

**Validation:** Seunghye Lee, Min Hye Kim, Se-Ho Chang, Hyun-Jung Kim.

**Pathological confirmation:** Min Hye Kim, Jong Sil Lee.

**Writing – original draft:** Sehyun Jung.

**Writing – review & editing:** Hani Jang.
